# Chlorodyne

**Published:** 1866-09

**Authors:** 


*Chlorodyne.*—We are in receipt of a Circular from Albert Ross & Co., Cincinnati, making known to the Profession the preparation, by Mr. E. A. Chandler, of "an article *fully equal, and in some respects superior*, to the English Chlorodyne."—The preparation contains, in thirty drops of the Chlorodyne, "one-eighth grain morphia, one quarter grain ext. cannabis, four drops chloroform and a small proportion of the oils peppermint and capsicum." We have tested, to some extent, the anodyne virtues of the Chlorodyne, from a specimen sent us by the above firm, and think favorably of it.

